# Transgenic validation of a promoter strongly inducible by *Agrobacterium tumefaciens*

**DOI:** 10.1038/s41598-025-30002-8

**Published:** 2025-12-01

**Authors:** Rakesh K. Sinha, Preeti Shakya, Rajendran K. Selvakesavan, Gregory Franklin

**Affiliations:** Institute of Plant Genetics of the Polish Academy of Sciences, Strzeszyńska 34, Poznań, 60-479 Poland

**Keywords:** Inducible promoter, *cis*-regulatory elements, *Agrobacterium tumefaciens*, *Hypericum perforatum*, Transgenic plants, Plant-pathogen interaction, Biotechnology, Cancer, Computational biology and bioinformatics, Microbiology, Molecular biology, Plant sciences

## Abstract

We report for the first time the isolation and functional characterization of a novel promoter inducible by *Agrobacterium tumefaciens*, the causative agent of crown gall disease, which leads to significant crop losses. Chemical control of this neoplastic disease is ineffective, since bacterial presence is not essential for T-DNA mediated tumor development. Moreover, *A. tumefaciens*-mediated transformation, a cornerstone of plant biotechnology, fails in many recalcitrant species due to poorly understood mechanisms. A unique 1086 bp promoter (HyPRO) sharing only ~ 7% similarity with known sequences in NCBI was isolated upstream of the *hyp1* gene from *Hypericum perforatum*. In silico analysis revealed multiple *cis* regulatory elements (CREs), including WRKY710S, W box, PALBOX, GT1, and VRE, associated with biotic stress responses. Transgenic tobacco plants expressing *β glucuronidase (GUS)* under HyPRO showed strong induction by *A. tumefaciens*, significantly higher than induction by *Pseudomonas syringae*. Upstream truncation of the promoter significantly reduced GUS expression, indicating essential regulatory elements lie upstream of position − 728. This *A. tumefaciens* responsive promoter offers a valuable tool to dissect plant defense and could enable innovative transgenic strategies for crop improvement and resistance to neoplastic diseases. Characterization of *A. tumefaciens*-specific CREs using the truncation approach is currently underway in our laboratory.

**Supplementary Information:**

The online version contains supplementary material available at 10.1038/s41598-025-30002-8.

## Introduction

*Agrobacterium tumefaciens* is a phytopathogen that can introduce its transferred DNA (T-DNA) into the plant genome. The T-DNA contains genes for the production of auxin, cytokinin and opines, but cannot be transcribed or translated within the bacterium. Once integrated into the plant genome, the production of auxin and cytokinin leads to uncontrolled cell division, causing crown gall disease, and the opines produced by the tumor serve as a good source for both nitrogen and carbon^1^. Crown gall disease affects a wide range of plant species^2^. Although it is very difficult to control the disease by chemical means, as the physical presence of the bacteria is not required for the development of the disease after T-DNA transfer, this natural process of gene transfer between kingdoms has been used to create transgenic plants and is an indispensable tool for modern functional genomics and plant improvement. Despite this, several economically important plant species, such as *Hypericum perforatum* remain recalcitrant to *A. tumefaciens-*mediated transformation, making it difficult to use genetic manipulation for functional studies^3^.

*A. tumefaciens* has been postulated not to induce host plant defense response^4^, since it is closely related to the symbiotic *Rhizobium*. The inability of the flagellins from this bacterium to induce plant defense response^5^ and the active suppression of host defense responses after the transfer of T-DNA complex^6^ support this hypothesis. Moreover, *A. tumefaciens* appears to manipulate the host plant’s cellular machinery in a way that promotes its own survival and proliferation without triggering a robust defense response^7–9^. In *H. perforatum*, phenolic oxidative coupling protein (*hyp1)* gene is up-regulated in response to *A. tumefaciens*, as revealed by previous studies^10^. Although the *hyp1* gene was originally considered essential for hypericin biosynthesis^11^, later studies have categorized this as a pathogenesis-related (*PR*) gene^12,13^. PR proteins are a diverse group of proteins that are induced in plants in response to pathogen infection and other stress factors. They are key components of the innate immune system of plants and play a role in defense against various pathogens and in promoting systemic resistance. Correspondingly, the induction of *hyp1* has been reported upon various biotic and abiotic stressors^10,12^. We also observed that *hyp1* expression was induced following treatment with *A. tumefaciens* (Fig. S1).

Plant promoters are DNA sequences that are located upstream of the transcription start site (TSS) of a gene which influence the timing, location and extent of gene expression. Promoters contain binding sites for transcription factors (TFs) such as *cis*-regulatory elements (CREs) that are crucial for the precise regulation of genes^14,15^. As the promoter controls the spaciotemporal expression of genes, investigating this region could provide crucial insights on the gene regulation and function. Promoter can be constitutive, driving continuous gene expression, or inducible, activating gene expression only under specific environmental or developmental condition. Inducible promoters are particularly valuable under biotic stress conditions, as they enable spatial and temporal regulation of transgene expression in response to pathogen attack or pest infestation. This targeted activation minimizes energy expenditure under non-stress conditions and reduces the risk of unintended metabolic burden or growth penalties often associated with constitutive expression^16^. Hence, inducible promoters serve as precise tools for fine-tuning plant defense responses, enhancing resistance without compromising overall plant fitness.

Although pathogen-inducible promoters such as pathogen-responsive *gn1* ^17^, *Sclerotinia sclerotiorum*-inducible *pBnGH17*^*D7*18^, *Magnaporthe grisea*-inducible *OsR2329*, *OsR2184*, and *OsPBZ1* ^19^, *Rhynchosporium secalis*-inducible *HvGER4c*^20^ have been identified, reports on promoters specifically responsive to *A. tumefaciens* are scarce. Previous studies have shown induction of host genes or localized activity of certain endogenous promoters during *A. tumefaciens* infection^21,22^, but no promoter has yet been isolated and functionally characterized as an *Agrobacterium*-inducible regulatory element. Here, we report the isolation and functional validation of a novel promoter from *Hypericum perforatum* (HyPRO) that exhibits strong inducibility by *A. tumefaciens*.

## Materials and methods

### Biological materials

*Hypericum perforatum* L. cultivar ‘Helos’ variety seeds used in the present study was purchased from Richters Herbs, 357 Highway, 47 Goodwood, ON L0C 1A0, Canada. The seeds of *Nicotiana tabacum* cultivar variety ‘Petit Havana SR1’ were obtained from the Department of Biology, University of Minho, Braga, Portugal. Plant pathogens *Pseudomonas syringae* pv. tabaci. *and Pectobacterium carotovorum subsp.* carotovorum were obtained from the Department of Virology and Bacteriology, Institute of Plant Protection – National Research Institute (IOR-PIB), Poznan.

### Promoter isolation

Seedlings of *H. perforatum* were grown in vitro as previously reported^23,24^ in half-strength Murashige and Skoog (MS) medium (Duchefa Biochemie, Netherlands)^25^ supplemented with 0.5 ppm indole-3-butyric acid (IBA, Sigma-Aldrich, USA). Genomic DNA was extracted from young leaves of *H. perforatum* using the GenElute™ Plant Genomic DNA Miniprep Kit (Sigma-Aldrich) according to the manufacturer’s instructions. The 5′ regulatory region was isolated using the random amplification of genomic ends (RAGE) method^26^. In brief, genomic DNA (2 µg) was digested in two independent reactions with the blunt-cutting restriction enzymes SspI and DraI (Thermo Fisher Scientific, Cat. No. ER0771 & ER0221) for 30 min at 37 °C and then purified with 3 M sodium acetate (1/10 volume) and three volumes of ice-cold ethanol, followed by a wash with 70% ethanol. The purified, digested DNA was ligated with an annealed adapter having a long arm and short arm (Table 1). The forward primer was designed from the upstream long arm of the adaptor and the reverse primer was designed from the *hyp1* gene sequence of *H. perforatum* from NCBI (accession number GU324244.1) (Table 1). DNA ligated with the adaptor was used as a template for primary PCR with the adaptor-specific forward primer (ADA1) and the gene-specific reverse primer (HYPGSP1) (Table 1). The reaction was set to 25 µl final volume with 2 mM dNTPs, 0.4 µM primers, 1 U *Pfu* DNA polymerase (Promega, Cat. No: M7741) and appropriate buffer. In addition, a nested PCR was performed using 1 µL of the 50 times diluted primary PCR product as template, 0.25 µM forward (ADA2) and reverse primers (HYPGSP2), 1.5 mM dNTPs, 1 U *Pfu* DNA polymerase and the corresponding buffer.

**Table 1:** Primers used for isolation and cloning of promoter.

Primer	Sequence (5′ − 3′)
LADAP (Long arm of the adapter)	CTAATACGACTCACATAGGGCTCGAGCGGCCGCCCGGGCAGGT
SADAP (Short arm of the adapter)	ACCTGCCC-NH_2_
ADA1 (Adapter specific forward primer 1)	GGATCCTAATACGACTCACTATAGGGC
ADA2 (Adapter specific forward primer 2)	AATAGGGCTCGAGCGGC
HYPGSP1 (Gene specific reverse primer 1)	GAAAGTGATTTTGGTGACGGTGCCGACA
HYPGSP2 (Gene specific reverse primer 2)	CGACACCTCCATCGCCTT
HYPGSP3 (Gene specific reverse primer 3)	CAACATGTACGTGAAGGGATGTCCATC
HYPGSP4 (Gene specific reverse primer 4)	CCTTCGACAATTTCGCCGC
PJET1-2 F (Forward primer for sequencing from cloning vector)	CGACTCACTATAGGGAGAGCGGC
PJET1-2R (Reverse primer for sequencing from cloning vector)	AAGAACATCGATTTTCCATGGCAG

### Promoter cloning and sequencing

The amplified product from the secondary nested PCR reactions was purified from the gel using GeneJET Gel Extraction Kit (Thermo Scientific, USA). Approximately 50 ng of the purified product was used for ligation in a 20 µL reaction using the Clonejet PCR cloning vector (pJET1.2/blunt cloning vector). The ligated plasmid was transformed into chemically competent *Escherichia coli* strain DH5α (Thermo Scientific, USA) *via* freeze-thaw method and plated onto Luria Bertani (LB) medium (Sigma, USA) containing selection antibiotic 100 mg/L ampicillin (Sigma, USA). Plasmids were isolated from 3 different colony PCR (using PJET1-2 F and PJET1-2R primers) positive colonies using the Plasmid Mini Prep Kit (Qiagen, Germany) and sequenced using the forward primer PJET1-2 F and the reverse primer PJET1-2R (Table 1). Sequencing was performed by a paid service of the Adam Mickiewicz University in Poznan. Alignment was performed using Clustal Omega^27^ to confirm the presence of upstream sequences from which the reverse primer for the RAGE experiment was designed.

***Cis*- regulatory element (CRE) analysis**.

Promoter was analyzed using Plant cis-acting regulatory DNA elements (PLACE; https://www.dna.affrc.go.jp/PLACE/?action=newplace), PlantCARE (https://bioinformatics.psb.ugent.be/webtools/plantcare/html/), and Plant Promoter Analysis Navigator (plantPAN 2.0; http://plantpan2.itps.ncku.edu.tw/index.html) software^28–30^.

### Plant expression vector construction

The promoter sequence was synthesized and cloned into the Gateway Cloning Entry Vector pGenDONR (GenScript, Netherlands). The vector was then transferred to competent *E. coli* (DH5α) cells using the freeze-thaw method and plated onto selection LB medium plates containing 50 mg/L kanamycin (Sigma, USA). The positive colonies were selected by colony PCR after overnight incubation. The plasmids from the positive colonies were isolated using the Plasmid DNA Mini Prep Isolation Kit (Qiagen, Germany) and sequenced as mentioned before to confirm the sequence similarity with the identified promoter sequences. The promoter was transferred by recombination from the entry vector (pGenDONR-HyPRO) into the destination binary vector pKGWFS7 (VIB, Ghent University, Ghent, Belgium). Recombination was performed with the Gateway™ LR Clonase™ II enzyme mix (Invitrogen, USA) according to the manufacturer’s protocol to produce the binary vector pKGWFS7-HyPRO. After recombination, the vector was transformed into competent *E. coli* (DH5α) cells using the freeze-thaw method and plated onto LB medium plates containing 100 mg/L spectinomycin (Sigma, USA). Positive colonies were identified using colony PCR and sequence confirmed as mentioned previously. Further to carryout deletion studies, selected part of the promoter was amplified by PCR to obtain 728 (HYP1TR1F and HYP1R primers, Table S1) and 488 base pairs (HYP1TR2F and HYP1R primers, Table S1), cloned and vectors for plant expression (pKGWFS7-HyPRO-TR1 and pKGWFS7-HyPRO-TR2) were constructed as described above.

### Generation of transgenic lines

*N. tabacum* seeds were first surface sterilized with 70% ethanol, followed by three washes with sterile water for 1 min each. Then the seeds were treated with 1.5% active chlorine equivalent sodium hypochlorite for 6 min and then washed three times with sterile distilled water for 1 min each. The washed seeds were plated on half-strength MS basal medium. The plates were kept at 25 °C in a tissue culture chamber with a photoperiod of 16 h light and 8 h darkness. The germinated seedlings were transferred to tissue culture flasks containing the same media for further growth.

*A. tumefaciens*-mediated tobacco transformation was performed according to the method described by Horsch et al. (1985)^31^ with some modifications. In brief, leaves of in vitro-grown plants were cut into slices using a sterile blade and pre-cultured overnight on MS basal medium plates. A single colony of *A. tumefaciens* strain EHA105 containing the binary pKGWFS7-HyPRO plasmid was transferred to 5 mL LB broth containing 50 mg/L spectinomycin and 20 mg/L rifampicin (Sigma, USA) and incubated in a rotary shaker set to 28 °C at 180 rpm for 24 h. The next day, 0.5 mL of the grown culture was inoculated into 100 mL medium and incubated as above. Once reached an optical density of 0.8–1.0 at 600 nm, the culture was centrifuged at 1240 x *g* (Eppendorf Centrifuge 5804, Rotor FA-45-6-30) for 10 min to pellet the bacteria. The bacterial pellet was gently re-suspended in MS basal medium and used for infection. The leaf discs precultured for a day were collected in a sterile Petri dish and infected with *A. tumefaciens* suspension for 10 min by gently swirling. After infection, the excess bacteria were removed using sterile blotting paper and transferred to the preculture plates and incubated at 25 °C in the dark for 2 days for co-cultivation. After two days or once the bacterial growth was visible around the tissues, the explants were washed with 500 mg/L ticarcillin disodium (Sigma, USA) and transferred to selection medium containing 1 mg/L 6-benzylaminopuryna (BAP, Sigma-Aldrich, USA) and 0.1 mg/L α-naphthaleneacetic acid (NAA, Sigma-Aldrich, USA) with 100 mg/L kanamycin and 200 mg/L ticarcillin disodium. The explants were subcultured to fresh selection medium containing 1 mg/L BAP and 0.1 mg/L NAA with 100 mg/L kanamycin every 15 days until shoot regeneration. Well-developed shoots were excised transferred to MS medium containing 100 mg/L kanamycin supplemented with 0.5 mg/L IBA for rooting. Rooted plantlets were transferred to the greenhouse for hardening. The transgenic tobacco plants were screened by PCR amplification (HYP1TR2F and HYP1R, Table S1). The seeds from T0 transgenic events were collected from self-pollinating plants to ensure the genetic makeup of the next generation is known. The seeds were germinated in half strength medium containing 100 mg/L kanamycin to observe the segregation. Positive plants were further grown in green house for collecting seeds. Homozygous plants were selected based on the segregation pattern shown by the T2 transgenic plants (Plants selected for which 100% seeds were germinated in the selection medium), following the protocol reported earlier^32^.

### Treatment conditions and gene expression analysis

Homozygous seeds of transgenic tobacco obtained were germinated in MS basal medium with 100 mg/L kanamycin. Two-week-old transgenic tobacco seedlings were treated with wild-type *A. tumefaciens* strain EHA105, *Pseudomonas syringae* pv. tabaci. and *Pectobacterium carotovorum subsp. carotovorum* in half-strength MS medium at 25 °C. The seedlings were kept in liquid, half-strength MS medium. The bacterial cultures were harvested by centrifugation, resuspended in half-strength MS medium, and added to the seedling cultures to a final optical density at 600 nm of 0.8. A corresponding volume of sterile, half-strength MS medium was used for mock inoculation. GUS assays were then performed at each time point with both the treated and mock-inoculated samples. After different time periods (1 h, 2 h and 4 h), the seedlings were removed from the bacterial solution and washed three times with distilled water and tested for the induction of the promoter. In another experiment seedlings were tested for promoter induction by flagellin and signaling molecules. A 2 mM stock of flagellin22 (GenScript, USA) salicylic acid (Sigma, USA) and nitric oxide donor (sodium nitroprusside; Sigma, USA) were prepared. As explained above, seedlings were independently treated with 20 µM flagellin22 (Flg22), 200 µM salicylic acid (SA) and 200 µM sodium nitroprusside (NO) for 0 h, 1 h, 2 h and 4 h, after which the seedlings were collected for GUS analysis. Seedlings subjected to the same procedure, but treated only with half-strength MS medium, served as control.

Histochemical GUS staining was performed for qualitative analysis of the promoter. Treated and control seedlings were transferred to 50 mL Falcon tubes and added with a solution containing 0.5 mg/mL X-Gluc, 20 mM phosphate buffer (pH 7.0), 10% Triton, 10 mM EDTA, 5 mM K_3_FeCN_6_ and 5 mM K_4_FeCN_6_. The tubes were wrapped with aluminum foil and incubated in the dark at 37 °C in a hot air oven. After 12 h, the solution was discarded and the seedlings were treated with 70% ethanol to remove the chlorophyll and photographed using a light microscope with camera. Prior to imaging, the shoots and roots of each seedling were separated using a sterile scalpel blade.

Quantitative real-time PCR (qRT-PCR) analysis of *GUS* gene was performed to quantify the promoter activity. Total RNA was isolated from the seedlings using InviTrap^®^ Spin Plant RNA Mini Kit (Invitek Molecular GmbH, Austria). First-strand cDNA was synthesized from 1 µg RNA using M-MLV Reverse Transcriptase (Promega, USA) following the manufacturer’s instructions. qRT-PCR was performed using SensiFAST SYBR No –ROX Kit (Meridian Bioscience, Cincinnati, USA) in a LightCycler 480 real-time PCR system (Roche, Basel, Switzerland) as reported earlier from our lab^24^. The expression of the *GUS* gene was normalized using *GAPDH*^33^ as reference gene and data were analyzed using delta-delta Ct method^34^. All the primers used are listed in supplementary information (Table S1).

### Statistical analysis

Each treatment comprised 3 replicates and the experiments were repeated at least three times. Data were analyzed by one-way analysis of variance (ANOVA) followed by Dunnet’s Multiple Comparison Test using GraphPad Prism software version 9 for Windows (California, USA).

## Results

### Isolation and cloning of the promoter

A nested PCR amplification with the primers ADAII and HYPGSP2 resulted in an amplicon containing the upstream region of the *hyp1* gene. Among the two restriction enzymes used, DraI yielded an amplicon size of 850 bp in nested PCR, and this fragment was further cloned and sequenced. The sequencing results showed 692 bp upstream of the gene along with 154 bp of the gene sequence. A genome walk upstream to this region was performed using reverse primers HYPGSP3 and HYPGSP4 (designed in the upstream region of the newly identified 692 bp promoter sequence) and forward primers ADAI and ADAII, which generated an 800 bp amplicon. An upstream sequence of 1086 bp, followed by the *hyp1* gene, was obtained after removal of the common sequences from both amplicons (Fig. S2).

***Cis*-regulatory elemental analysis**.

**Fig. 1:**
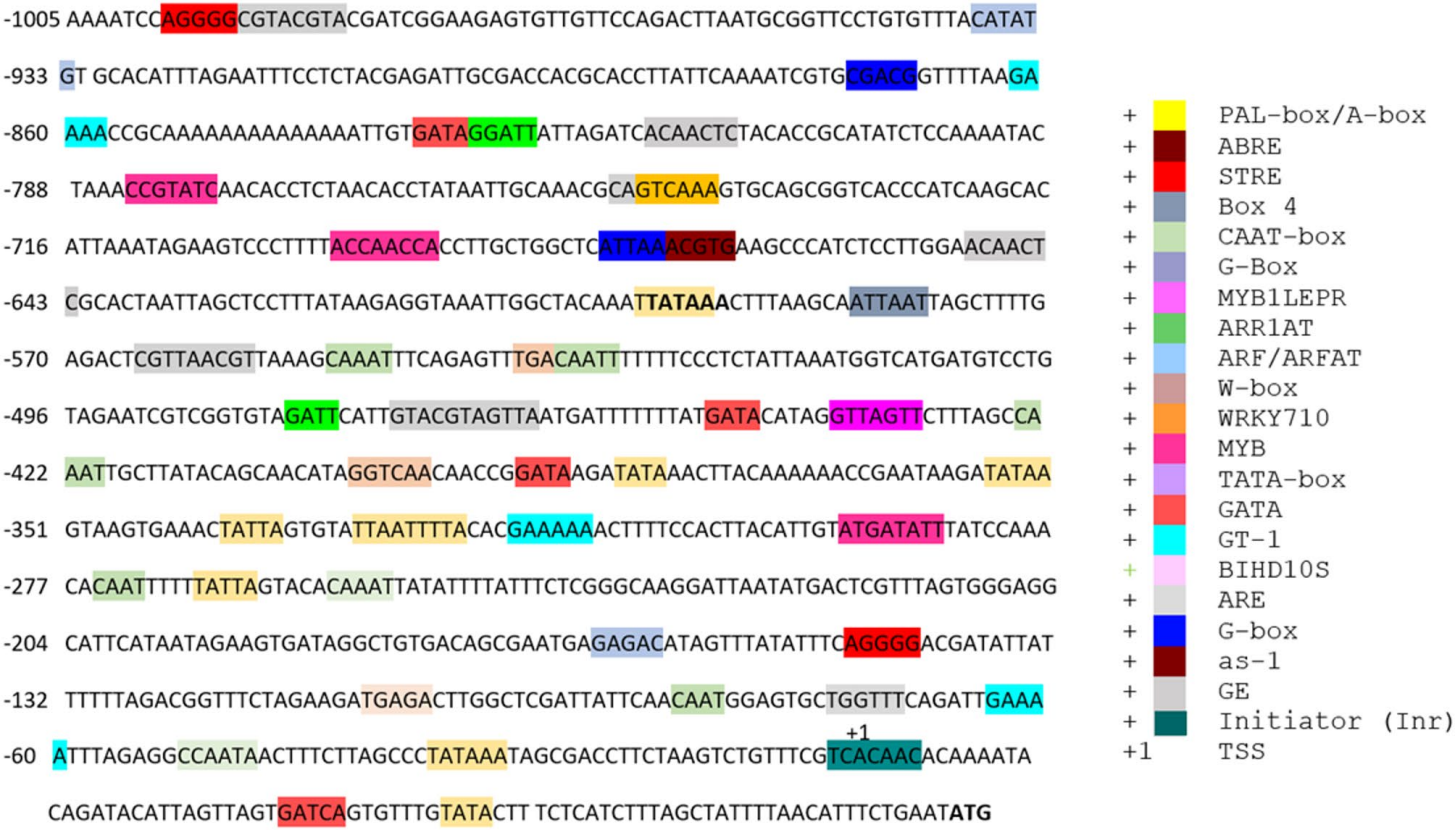
Nucleotide sequence of HyPRO showing the CRE*s* identified by PlantCARE, PLACE and PlantPAN tools.

The BLAST results of the promoter sequence showed no significant homology in NCBI database (Fig. S3). The CREs of the 1086 bp promoter sequence upstream of ATG (translation initiation site ATG is considered + 1) were predicted by searching in PLACE, plantPAN 2.0 and PlantCARE database (Fig. 1). Transcription start site was at the 81 bases upstream of the start codon and marked as + 1. This assignment is supported by alignment of the NCBI accession AY148090.1 cDNA derived from a λ-TriplEX2 library^11^ that selectively captures capped, full-length transcripts to our 1,086 bp upstream genomic fragment, and by the presence of a consensus TATA-box motif centered 26 bases upstream of the mapped + 1 position. The promoter analysis confirmed the presence of various types of CREs in the nucleotide sequence, the major CREs are of plant defense, biotic stress, environmental stress response, phytohormones response, light response, tissue/cell specificity and elements related to transcription factor binding sites (Table 2, Table S2). A number of CREs were found throughout promoter sequence, including those involved in the response to pathogen infection, wounding, hormones like gibberellin, abscisic acid, cytokines, auxin, methyl-jasmonate (MeJA), ethylene, and SA. Moreover, various key transcription factor binding sites are present such as EEF/AP2, MYB, NAC, WRKY, bZIP, Dof, and C2H2 (refer to Table S2 for more details).

**Table 2:** Putative CREs of HyPRO predicted to be involved in biotic stress.

CREs	Position	Function	HyPRO	HyPRO-TR1	HyPRO-TR2
W-box	−606	Wound-responsive	1	1	0
WRKY710S	−827, −813, −610 −583, −482, −813, −303, −259, −83	Biotic stress and GA responsive	9	6	4
WBOXNTERF3	−288, −302, −532, −633, −813	Wound-responsive	5	1	0
BIHD1OS	−259, −610	Response to pathogen attack	2	2	2
ASF1MOTIFCAMV, AS−1	−84	Pathogen inducible, Auxin and SA responsive	1	1	1
CGACG OSAMY3	−956	Disease resistance responsive	1	0	0
PALBOXPPC	−781	Phenylpropanoid biosynthesis and wound-inducible peroxidase responsive element	2	0	0
BOXLCOREDCPAL	−777	Fungal elicitor responsive	1	0	0
GT-1	−940	Pathogen responsive	1	0	0
VRE	-304	Group I bZIP protein, VIP1 binding site, VIP1 activate PR protein	1	1	1
STRE	−1079, −228	Stress responsive	2	1	1
TC-rich repeats	−949	Plant defense responsive	1	0	0
TATABOXOSPAL	−796	Plant defense responsive	1	0	0
MYB1LEPR	−520	Plant defense responsive	1	1	0
WBBOXPCWRKY1	−828	Pathogen/elicitor responsive	1	0	0

0, denotes absence of CREs.

***GUS*** **gene expression under the control of full-length and truncated promoter**.

**Fig. 2:**
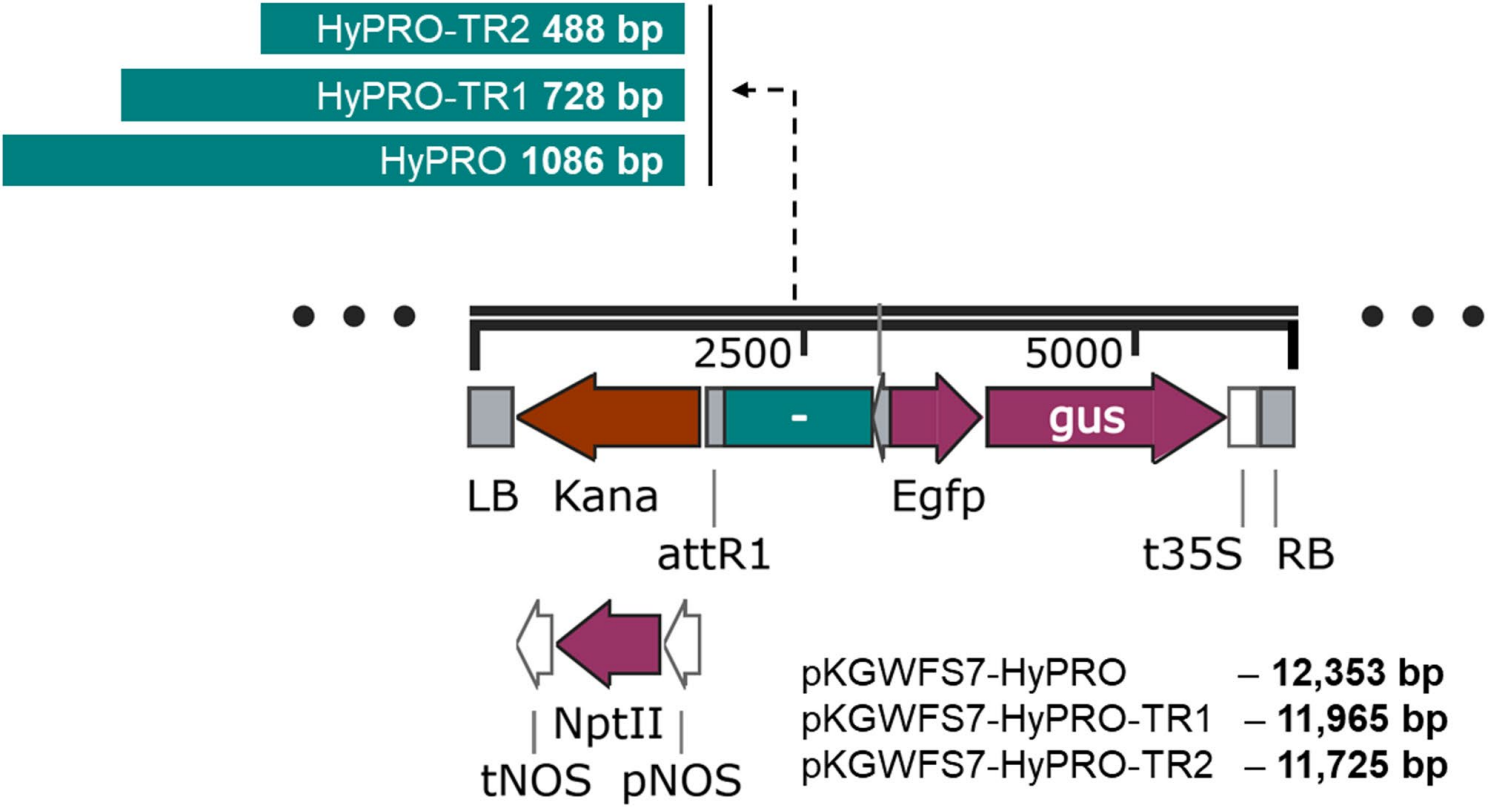
Schematic representation of the full-length and truncated promoter constructs used to generate transgenic plants.

Based on the findings from the prediction of CREs in the promoter sequence located at the distal end of the promoter, we examined the upstream fragment ranging from 480 to 1086 bp and found that it includes multiple key components linked to stress responses and transcription factor binding sites. To assess the function of the distal end of the promoter, we have generated two truncated promoter fragments of 728 bp (HyPRO-TR1, −1 to −728) and 488 (HyPRO-TR2, -1 to − 488) bp (Fig. 2, Fig. S2).

**Fig. 3:**
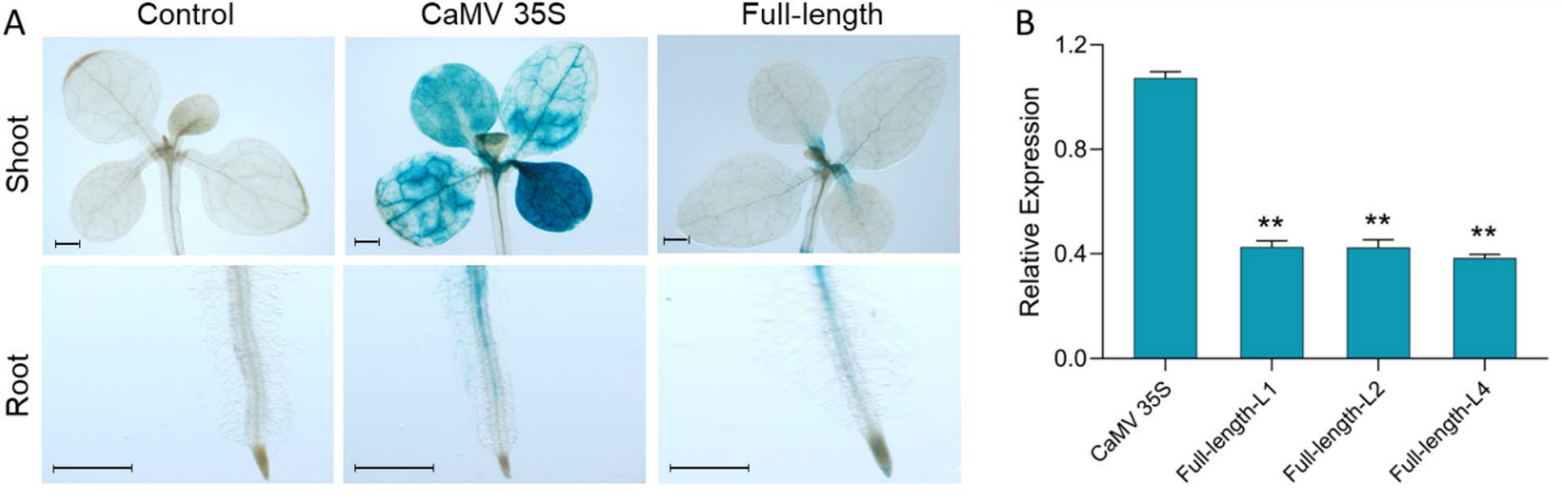
(**A**) GUS assay of transgenic tobacco seedlings expressing the *GUS* gene under the control of CaMV 35S promoter and HyPRO (scale = 1 mm); Prior to imaging, the shoots and roots of each seedling were separated using a scalpel blade. (**B**) qRT-PCR analysis of the *GUS* gene expressed in transgenic tobacco seedlings under the control of the CaMV 35S and three lines of HyPRO (L1, L2 and L4) with a minimum of three samples each line. Results are expressed as mean ± SE. (Dunnett’s multiple comparison test; ** *P* < 0.01).

To functionally validate the promoter identified through in silico analysis, we generated transgenic lines in which the *GUS* reporter gene was placed under the control of HyPRO, HyPRO-TR1, or HyPRO-TR2, and subsequently used these plants in induction assays to evaluate promoter responsiveness to *A. tumefaciens* and other elicitors. We conducted histochemical staining to test the promoter activity in transgenic lines (Fig. 2). The GUS histochemical staining of transgenic tobacco indicated that the promoter showed reduced activity in expressing GUS compared to CaMV 35S. Moreover, the results also indicated truncation of the upstream region of the identified promoter sequence further reduces the promoter activity (Fig. 3). Moreover, truncation of the upstream region of the promoter sequence further reduced GUS expression (Fig. 3), indicating that the 390 bp distal end fragment contains important CREs that can act as enhancers, modulating the transcriptional activity of *hyp1* gene. In accordance with the typical characteristics of eukaryotic promoters, typical core elements, TATA-box and CAAT-box (TSS) (Fig. 1; Table 2) were present in both truncated and full-length promoter. However, the absence of vital CREs in truncated promoter, such as one GT-1 element, seven pathogen-responsive elements, two abscisic acid response elements, two gibberellin response elements, one salicylic acid response element, four cytokinin response elements, one auxin response element, and one STRE response element (Table 2, Table S3) might contribute to the decreased expression of the truncated promoter.

To understand the expression pattern of the promoter, GUS staining was performed with control and transgenic plants and their expression was compared with that of Cauliflower Mosaic Virus (CaMV) 35S, a constitutive promoter. GUS expression under the control of HyPRO was much lower compared to the CaMV 35S promoter and was restricted to the petiole and root of the seedlings (Fig. 3A). This result was consistent with the gene expression in the three different promoter lines. The qRT-PCR analysis revealed that *GUS* transcripts were abundant in the CaMV 35S lines, while very low levels of transcripts were found in the three lines expressing the *GUS* gene under the control of HyPRO (Fig. 3B).

***A. tumefaciens*** **strongly induces the promoter HyPRO**.

**Fig. 4:**
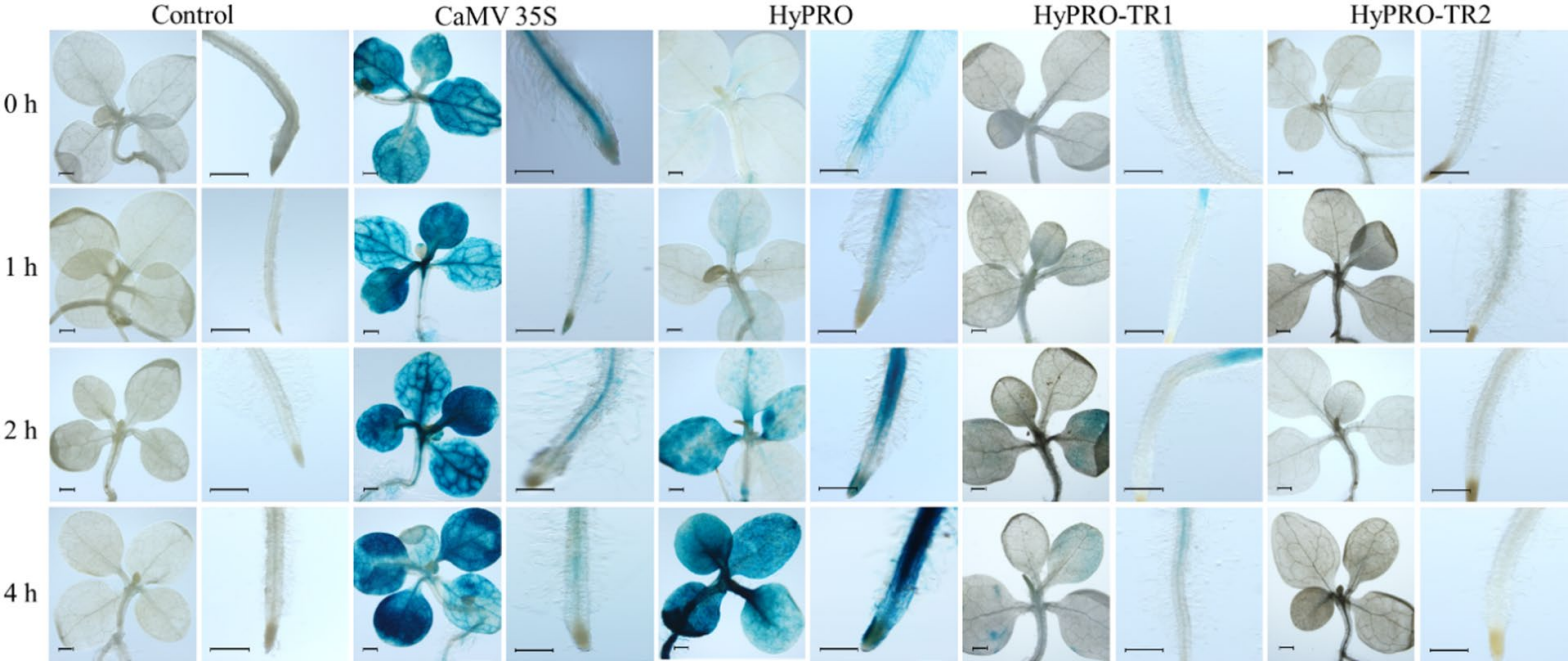
GUS assay of transgenic tobacco seedlings containing the *GUS* gene under the control of CaMV 35 S, HyPRO and truncated promoters (HyPRO-TR1 and HyPRO-TR2) after 0 h, 1 h, 2 h and 4 h of co-cultivation with *A. tumefaciens* (scale = 1 mm) compared to non-transgenic control.

The ability of *A. tumefaciens* to induce the promoter was evaluated by the GUS activity linked to the promoter. The GUS activity was monitored after 1 h, 2 h and 4 h after co-cultivation of transgenic seedlings with *A. tumefaciens*. The expression of *GUS* gene under the control of full-length promoter in transgenic seedlings treated with *A. tumefaciens* was similar to that in uninfected controls after 1 h of infection. However, after 2 h and 4 h of treatments, there was an increase in GUS activity in the shoot and root of the transgenic seedlings compared to control (Fig. 4).

The effects of promoter truncation on the expression of the GUS upon treatment with *A. tumefaciens* were examined using the two truncated promoters HyPRO-TR1 and HyPRO-TR2 (Fig. 4) and compared within the treatments for the same time points. The results showed that the truncation significantly impaired the inducibility of the promoter by *A. tumefaciens*. Compared to HyPRO-TR1, which showed weak GUS expression at later time points, HyPRO-TR2 showed no GUS expression in response to *A. tumefaciens*.

**Fig. 5:**
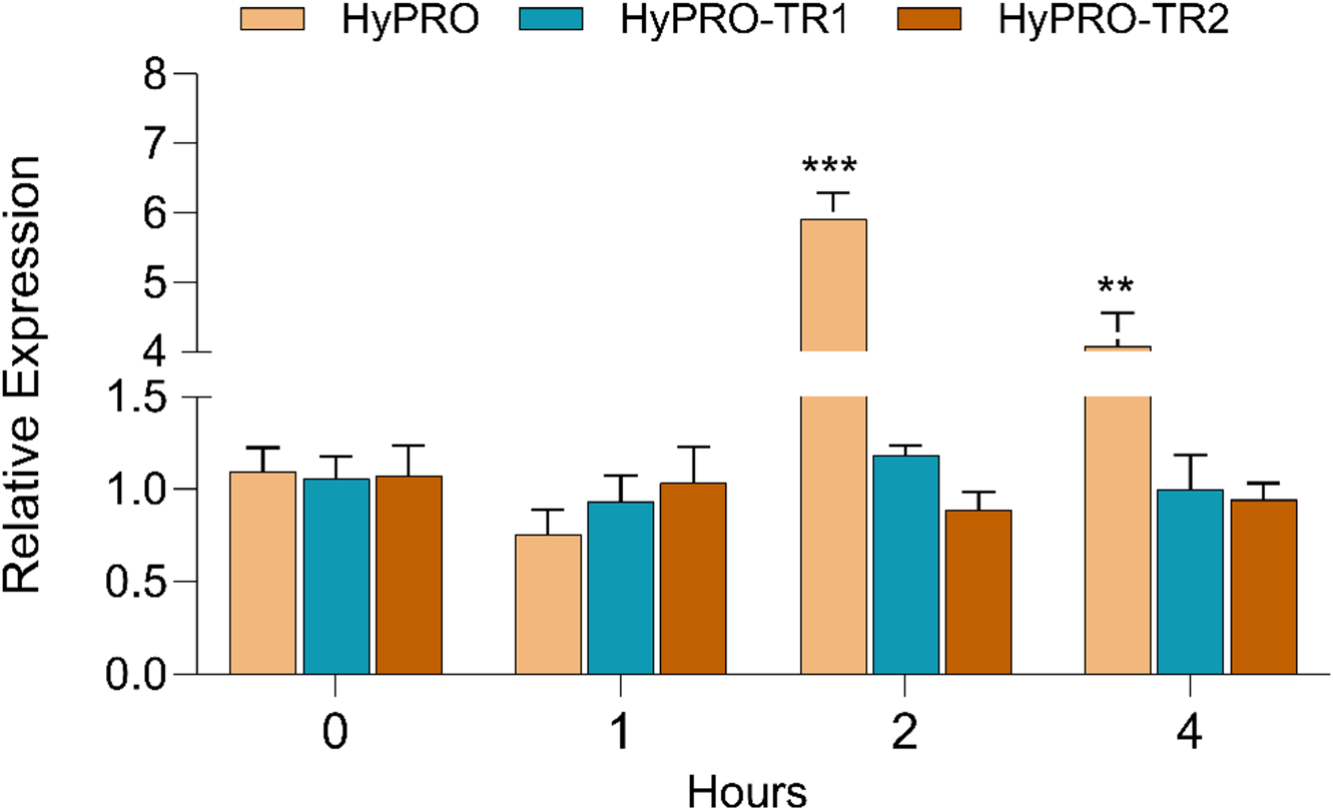
Relative expression of the *GUS* gene under the control of the full-length and, truncated promoters HyPRO-TR1 and HyPRO-TR2 after co-cultivation with *A. tumefaciens* in tobacco seedlings. Results are expressed as mean ± SE. Asterisks indicate statistically significant differences between treatment groups at the same time point. (Dunnett’s multiple comparison test; *** *P* < 0.001, ** *P* < 0.01).

Furthermore, the results from qRT-PCR indicated no notable change in *GUS* gene expression under the control of full-length promoter after 1 h (0.76 ± 0.14) of treatment with *A. tumefaciens*, while a significant increase was recorded at both 2 h (5.91 ± 0.37; *P* < 0.001, Dunnett’s test) and 4 h (4.07 ± 0.49; *P* < 0.01, Dunnett’s test) compared to the control. In contrast, the truncated promoter HyPRO-TR1 exhibited similar *GUS* gene expression after 4 h (1.00 ± 0.18) and 2 h (0.93 ± 0.13) compared to 1 h (1.18 ± 0.05) of treatment with *A. tumefaciens* (Fig. 5). Another truncated promoter HyPRO-TR2 have shown little or no effect of *A. tumefaciens* and shown consisted expression at all the time points 0 h, 1 h, 2 h and 4 h (1.07 ± 0.16, 1.03 ± 0.19, 0.89 ± 0.08, 0.94 ± 0.09). Reduced GUS expression observed in plants carrying the truncated promoters suggests that the regulatory elements required for optimal *GUS* gene expression are missing in these truncated promoters. Although the results suggest that the CRE/CREs in − 728 to − 1086 bp are essential for promoter activity induced by *A. tumefaciens*, their exact position need to be verified by further truncation or mutation experiments, which will be the focus of our future work.

**Fig. 6:**
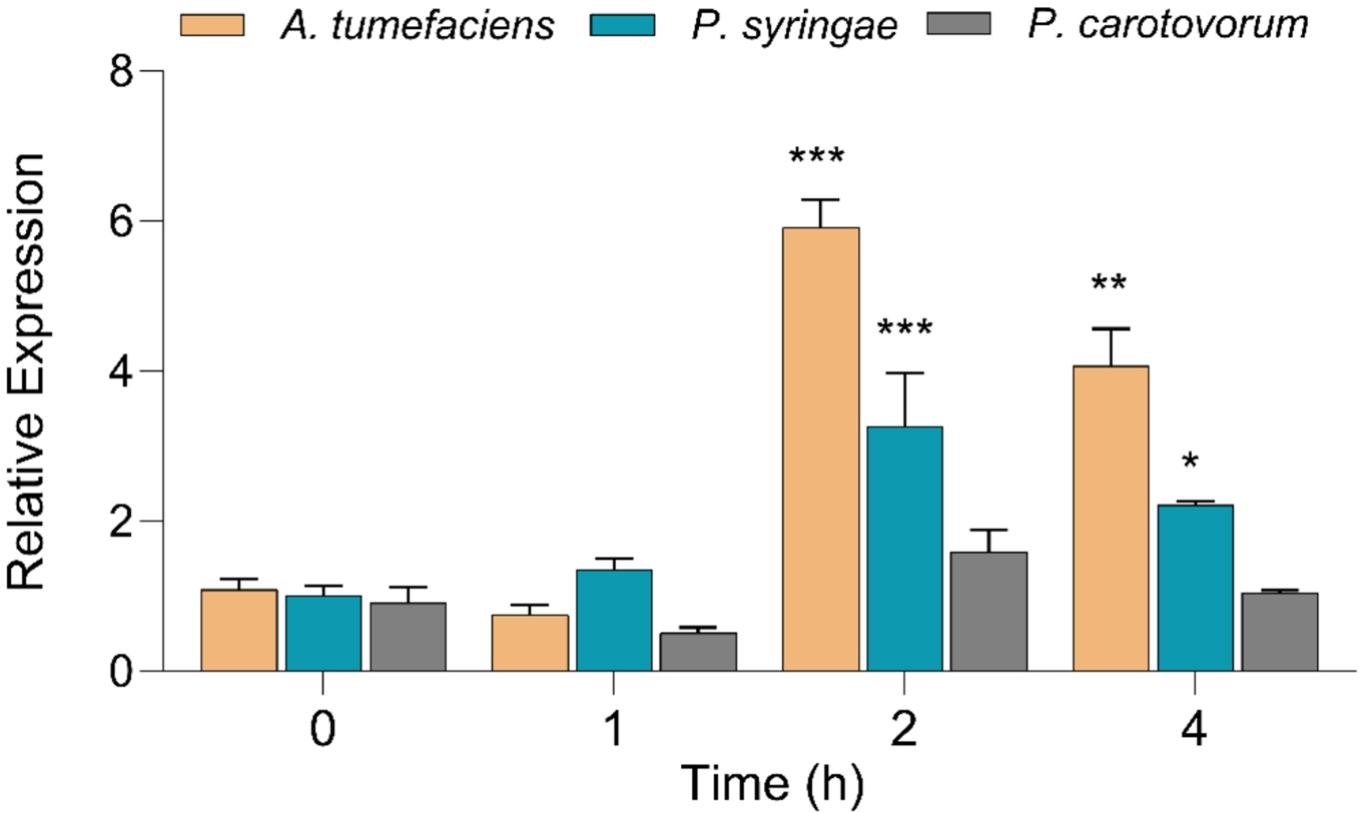
Relative expression of the *GUS* gene under the control of the full-length promoter HyPRO after co-cultivation with *A. tumefaciens*, *P. syringae* and *P. carotovorum* for 0 h, 1 h, 2 h and 4 h. The results are given as mean ± SE. The number of asterisks on a bar indicates the significance level of the difference between treatments at each time point as determined by Dunnett’s Multiple Comparison Test (*** *P* < 0.001, ** *P* < 0.01, * *P* < 0.05). The data for *A. tumefaciens* are the same as in Fig. 5 and were repeated for comparison purposes only.

To find out whether other pathogens can induce the promoter, we tested *P. syringae* and *P. carotovorum*. When the seedlings were co-cultivated with *P. syringae* and *P. carotovorum* in the same way as for *A. tumefaciens*, the relative *GUS* expression in response to *P. syringae* was increased 3.20 ± 0.71-fold (*P* < 0.001, Dunnett’s test) after 2 h compared to the control (1.01 ± 0.13), while the expression in response to *P. carotovorum* was not significantly altered (1.60 ± 0.29) (Fig. 6). While after 4 h of treatment the *GUS* expression in response to *P. syringae* was still significant higher (2.22 ± 0.06; *P* < 0.05, Dunnett’s test) while *P. carotovorum* was not significantly altered (1.05 ± 0.03).

### Flg22, SA and NO mildly induced the promoter HyPRO

**Fig. 7:**
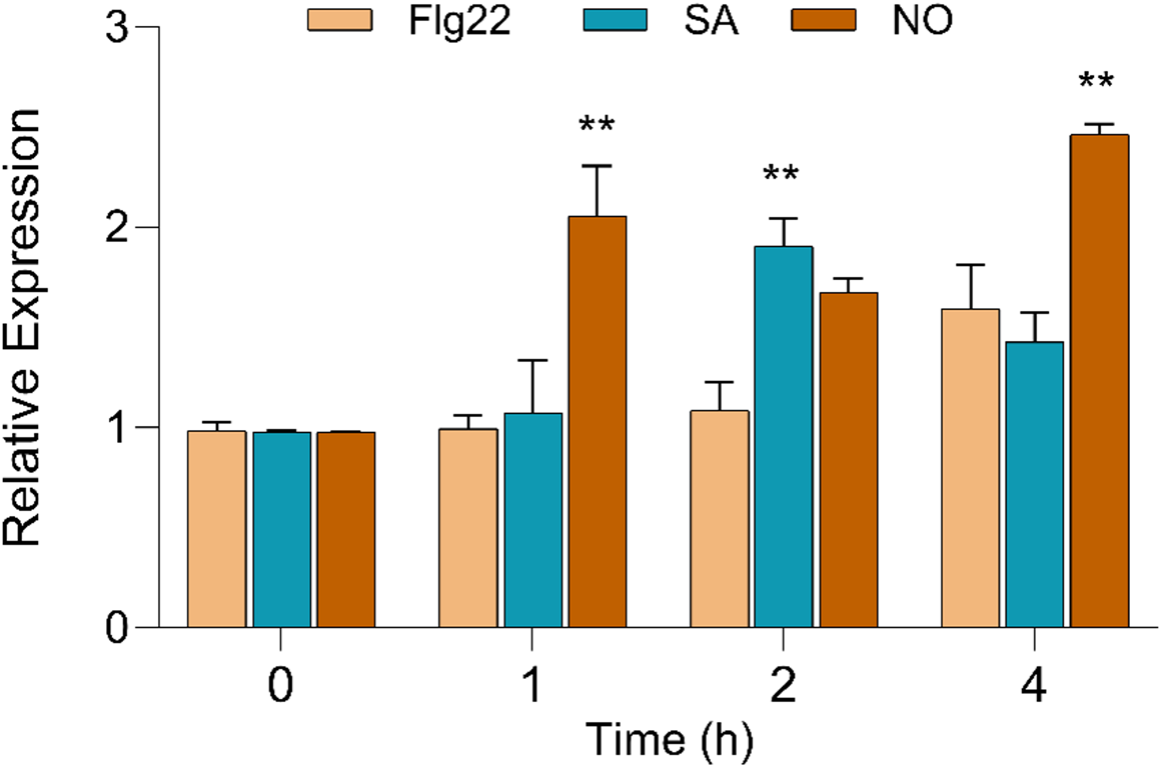
Expression of *GUS* gene after treatment with flagellin (Flg22), salicylic acid (SA), and sodium nitroprusside (NO) as revealed by qRT-PCR. Results are expressed as mean ± SE. Asterisks indicate statistically significant differences between treatment groups at the same time point. (Dunnett’s multiple comparison test; ** *P* < 0.01, * *P* < 0.05).

In addition to pathogen challenge, we also tested the effect of well-known defense-related signaling molecules and elicitors, including Flg22 (a bacterial flagellin-derived peptide recognized by plant pattern recognition receptors), SA (a key signaling molecule in systemic acquired resistance), and NO (a signaling molecule involved in plant defense). The expression analysis of the *GUS* gene showed that nitric oxide (NO) treatment could significantly induce the promoter by 2.05 ± 0.25 (*P* < 0 0.01, Dunnett’s test) and 2.40 ± 0.05 (*P* < 0.01, Dunnett’s test ) fold after 1 and 4 h of treatment, respectively, while SA showed 1.90 ± 0.14-fold (*P* < 0.01, Dunnett’s test) higher *GUS* gene expression than the control after 2 h of treatment (Fig. 7) and slightly reduced after 4 h (1.40 ± 0.15). On the other hand, no significant difference was observed after Flg22 treatment.

## Discussion

Promoters are DNA sequences that contain CREs to which TFs bind and regulate the expression of genes. The expression and functions of the TFs are influenced by a variety of transcriptional, post-transcriptional, and post-translational controls as they serve as primary regulators of plant defense^35^. These TFs or regulatory proteins when bound to the CREs can activate various defense and biosynthetic pathways related genes.

The analysis of the promoter nucleotide sequence has identified several vital CREs involved in pathogen responses, including TGA elements, W-boxes, ABRE, GT-1, AS-1, BIHD10S, and VRE. To regulate pathogen response, the W-box (TTGAC), TGACG motif and E-box (CANNTG) are vital CREs found in plant defense-related genes recognized by WRKY, basic leucine zipper (bZIP) and basic helix-loop-helix (bHLH) transcription factor families^36^. W-boxes are vital for activating *PR*-2 in *Arabidopsis thaliana* during pathogen attacks^37^; WRKY4 binds to the W-box of WRKY4 promoter to confer resistance to pathogen infection in rice^36^. Such specific promoters might control the expression of genes involved in the production of antimicrobial compounds, reinforcement of the plant cell wall, and the induction of defense-related genes. These responses collectively enhance the plant’s ability to resist and combat bacterial infections^38^. GT-1, AS-1 elements, recognized by GT-1 and AS-1 transcription factors, regulate defense gene expression during pathogen infections and involve in plant immunity^36,39^.

GUS expression was predominantly localized in the roots and petiolar region, consistent with previous reports of *hyp1* expression in the roots of *H. perforatum* seedlings^40^. The reduction in GUS expression in the roots of HyPRO-TR1 and HyPRO-TR2 seedlings is probably attributed to the absence of STRE in the distal fragments of these truncated promoters. Earlier reports suggest that the presence of STREs in promoters has been shown to enhance the expression of stress-responsive genes, thereby facilitating the plant’s adaptive responses^41^. The interplay between STREs and TFs such as MYB and WRKY is crucial, as these factors can enhance the recruitment of RNA polymerase II to the promoter, facilitating the transcription of downstream genes involved in stress tolerance^42,43^. Moreover, the spatial arrangement of STREs within the promoter region can influence the binding affinity of these TFs, further modulating gene expression. Studies have shown that the proximity of STREs to the core promoter region can significantly affect the overall transcriptional output under stress conditions^44^. No detectable GUS expression was observed in HyPRO-TR2 seedlings under either control or induced conditions, suggesting that this construct is transcriptionally inactive. While low basal GUS expression is present in HyPRO-TR1 seedlings, suggesting that HyPRO-TR1 is transcriptionally active, treatment with *A. tumefaciens* does not result in an increase in seedling GUS activity, suggesting that HyPRO-TR1 reduces the overall strength of the promoter and also abolishes inducibility.

When treated with *A. tumefaciens*, the expression of GUS slowly spread to other areas of the seedlings and ubiquitous after 4 h. The rapid switch from tissue-specific basal expression to widespread induction of HyPRO following *Agrobacterium* treatment likely reflects activation of transcription factors that respond to stress by activating stress-inducible *cis*-elements and associated signaling cascades that expand promoter activity under biotic stress^45^. The presence of VIP1 response elements (VREs) is crucial for how plants interact with *A. tumefaciens* and is vital for the nuclear import of VirE2 during the initial stages of T-DNA expression^46^. This mechanism triggers the defense responses of the plant, affecting the cellular processes of the host. As previously demonstrated, the *A. thaliana* VIP1 protein engages with *A. tumefaciens*, leading to the activation of a synthetic promoter that includes several copies of VRE. Earlier, VRE was identified as inducible upon *Agrobacterium* infection and mutation in the VRE almost completely disrupt VIP1 binding and transcriptional activation^47^. It establishes direct interactions with VREs in both in vitro and in vivo^48^. Such activation leads to a series of downstream signaling events that enhance the plant’s ability to respond to pathogenic threats. The qRT-PCR analysis revealed a rapid transcriptional induction of the HyPRO promoter, peaking at 2 h post-induction. In contrast, histochemical GUS staining, which reflects the cumulative activity of the stable GUS enzyme, showed the most intense signal at the later 4 h time point. This temporal pattern is consistent with the expected lag between mRNA transcription and the accumulation of the protein product. Our findings support this mechanism, as the early transcript surge detected by qRT-PCR at 2 h post-infection likely precedes the detectable accumulation of GUS protein, resulting in the widespread staining observed at 4 h (Figs. 4 and 5). Interestingly, HyPRO showed differential responses to other bacterial pathogens. *A. tumefaciens* treatment resulted in a significantly higher level of induction compared to the tobacco pathogen *P. syringae*^49^, while *P. carotovorum* exhibited no change in expression (Figs. 5 and 6). *Agrobacterium* infection induces salicylic acid signaling^50^, a pathway also triggered by *P. syringae*, a hemibiotrophic pathogen that activates SA-dependent defense responses and may thereby explain its ability to activate HyPRO^51^. In contrast, the necrotrophic pathogen *P. carotovorum* relies largely on cell wall–degrading enzymes to infect host tissues and may not activate the same defense signaling cascades^52^.

NO is a versatile gaseous signaling molecule that plays pivotal roles in plant growth, development, and defense responses. In the present study, NO treatment led to a significant increase in *GUS* gene expression, suggesting that NO plays an active role in modulating transcriptional responses through the promoter. The early and sustained gene expression observed in response to NO treatment may be attributed to redox mediated modulation of TFs, particularly WRKY, TGA, and MYB, whose target binding sites (W box, AS 1, and MYB motifs, respectively) are present in our promoter, suggesting that NO signaling likely enhances transcription by altering transcription factor DNA binding activity and facilitating RNA polymerase II recruitment^53^. Induced expression after NO treatment was also observed in the promoter of the pepper pathogen-induced membrane protein gene CaPIMP1^54^. Nonexpresser of PR (NPR) proteins are recognized as key receptors for SA and are known to interact with TGACG motif-binding (TGA) transcription factors^55,56^. Our observation of full-length promoter activation by SA is consistent with prior evidence showing that SA enhances the binding activity of TGA TFs, particularly TGA2 and TGA3, to as-1–type *cis*-elements through an NPR1-dependent mechanism. Moreover, the presence of TGA binding motif at -84 bp upstream of the start site might be a CREs, which allows SA-activated TFs to bind and boost gene expression^57^. Activation of TGA binding motif leads to the upregulation of defense-related genes, especially those influenced by the NPR1 protein, whose expression is stimulated by SA ^59^. In many defense responses, NO functions either upstream or in parallel with SA, contributing to the transcriptional activation of *PR* genes^59^. The stronger response of the full-length promoter to NO compared to SA might suggest that NO is a more immediate or dominant signal in triggering its activation, possibly by priming or enhancing SA signaling components like NPR1 and TGA. Flg22, a 22-amino acid peptide derived from bacterial flagellin, triggers a strong immune response in plants^60^. In our study, treatment with Flg22 resulted in only a low induction of the promoter, whereas a higher activation was observed with SA and NO. Taken together, these results position HyPRO as a defense-inducible promoter that responds to NO- and SA-mediated signaling cascades, and provide insights into the regulatory mechanisms underlying *Agrobacterium*-induced gene expression.

## Conclusions

In summary, our study has identified a novel plant promoter that can be induced by *A. tumefaciens*. Although this bacterium is recognized as a tool for plant transformation, it is a potent pathogen that causes neoplastic diseases in a wide range of plant species, leading to significant economic losses worldwide. Since the disease progresses after the initial transformation event regardless of the pathogen, it is imperative that strategies to eliminate *A. tumefaciens* are applied prior to infection. The promoter shown in the present study will be useful for the control of neoplastic disease when combined with appropriate genes that can protect against *A. tumefaciens* infection. Further studies are needed to determine the motifs associated with the induction of the promoter by *A. tumefaciens*. Experiments in this direction are underway in our laboratory.

## Supplementary Information

Below is the link to the electronic supplementary material.

Supplementary Material 1

Supplementary Material 2

## Data Availability

The dataset generated (Promoter sequence) during the current study is available in the NCBI GenBank repository (Accession No. PV876988).

## References

[1] Lang, J. et al. Fitness costs restrict niche expansion by generalist niche-constructing pathogens. *ISME J.* **11**, 374–385 (2017). 27801902 10.1038/ismej.2016.137 PMC5270578

[2] Gelvin, S. B. *Agrobacterium*-Mediated plant transformation: the biology behind the Gene-Jockeying tool. *Microbiol. Mol. Biol. Rev.* **67**, 16–37 (2003). 12626681 10.1128/MMBR.67.1.16-37.2003 PMC150518

[3] Hou, W., Shakya, P. & Franklin, G. A perspective on *Hypericum perforatum* genetic transformation. *Front. Plant Sci.* **7**, 879 (2016). 27446112 10.3389/fpls.2016.00879 PMC4919345

[4] Ditt, R. F., Nester, E. W. & Comai, L. Plant gene expression response to *Agrobacterium* tumefaciens. *Proc. Natl. Acad. Sci. U S A*. **98**, 10954–10959 (2001). 11535836 10.1073/pnas.191383498 PMC58580

[5] Felix, G., Duran, J. D., Volko, S. & Boller, T. Plants have a sensitive perception system for the most conserved domain of bacterial Flagellin. *Plant J.* **18**, 265–276 (1999). 10377992 10.1046/j.1365-313x.1999.00265.x

[6] Veena., Jiang, H., Doerge, R. W. & Gelvin, S. B. Transfer of T-DNA and vir proteins to plant cells by *Agrobacterium* tumefaciens induces expression of host genes involved in mediating transformation and suppresses host defense gene expression. *Plant J.* **35**, 219–236 (2003). 12848827 10.1046/j.1365-313x.2003.01796.x

[7] Gohlke, J. & Deeken, R. Plant responses to *Agrobacterium tumefaciens* and crown gall development. *Front Plant. Sci* **5**, (2014). 10.3389/fpls.2014.00155 PMC4006022 24795740

[8] Brown, P. J. B., Chang, J. H. & Fuqua, C. *Agrobacterium tumefaciens*: a transformative agent for fundamental insights into Host-Microbe Interactions, genome biology, chemical Signaling, and cell biology. *J. Bacteriol.* **205**, e00005–23 (2023). 36892285 10.1128/jb.00005-23 PMC10127608

[9] Etminani, F., Harighi, B., Bahramnejad, B. & Mozafari, A. A. Antivirulence effects of cell-free culture supernatant of endophytic bacteria against grapevine crown gall agent, *Agrobacterium tumefaciens*, and induction of defense responses in plantlets via intact bacterial cells. *BMC Plant. Biol.* **24**, 104 (2024). 38336608 10.1186/s12870-024-04779-1 PMC11297725

[10] Košuth, J., Hrehorová, D., Jaskolski, M. & Čellárová, E. Stress-induced expression and structure of the putative gene hyp-1 for hypericin biosynthesis. *Plant. Cell. Tissue Organ. Cult. (PCTOC)*. **114**, 207–216 (2013).

[11] Bais, H. P., Vepachedu, R., Lawrence, C. B., Stermitz, F. R. & Vivanco, J. M. Molecular and biochemical characterization of an enzyme responsible for the formation of hypericin in St. John’s wort (*Hypericum perforatum* L). *J. Biol. Chem.* **278**, 32413–32422 (2003). 12799379 10.1074/jbc.M301681200

[12] Karppinen, K., Derzsó, E., Jaakola, L. & Hohtola, A. Molecular cloning and expression analysis of hyp-1 type PR-10 family genes in *Hypericum perforatum*. *Front. Plant. Sci.* **7**, 526 (2016). 27148343 10.3389/fpls.2016.00526 PMC4838893

[13] Sliwiak, J., Dauter, Z. & Jaskolski, M. Crystal structure of Hyp-1, a *Hypericum perforatum* PR-10 Protein, in complex with melatonin. *Front Plant. Sci* **7**, (2016). 10.3389/fpls.2016.00668 PMC4870859 27242869

[14] Hu, M. et al. Comparative analysis of the LEA gene family in seven Ipomoea species, focuses on sweet potato (*Ipomoea Batatas* L). *BMC Plant. Biol.* **24**, 1256 (2024). 39725899 10.1186/s12870-024-05981-x PMC11670493

[15] Yang, X. et al. Genome-wide identification and characterization of bZIP gene family explore the responses of PsebZIP44 and PsebZIP46 in *Pseudoroegneria Libanotica* under drought stress. *BMC Plant. Biol.* **24**, 1085 (2024). 39548382 10.1186/s12870-024-05809-8 PMC11568582

[16] Kauder, F. et al. Expression of a modified Avr3a gene under the control of a synthetic pathogen-inducible promoter leads to *Phytophthora infestans* resistance in potato. *Plant. Biotechnol. J.* **23**, 1683–1701 (2025). 40059336 10.1111/pbi.14615 PMC12018830

[17] Kooshki, M., Mentewab, A. & Stewart, C. N. Pathogen inducible reporting in Transgenic tobacco using a GFP construct. *Plant Sci.* **165**, 213–219 (2003).

[18] Lin, L. et al. The sclerotinia sclerotiorum-inducible promoter pBnGH17D7 in *Brassica napus*: isolation, characterization, and application in host-induced gene Silencing. *J. Exp. Bot.* **73**, 6663–6677 (2022). 35927220 10.1093/jxb/erac328 PMC9629790

[19] Sasaki, K. et al. Characterization of two rice peroxidase promoters that respond to blast fungus-infection. *Mol. Genet. Genomics*. **278**, 709–722 (2007). 17805575 10.1007/s00438-007-0286-1

[20] Himmelbach, A. et al. Promoters of the barley Germin-Like GER4 gene cluster enable strong transgene expression in response to pathogen attack. *Plant. Cell.* **22**, 937–952 (2010). 20305123 10.1105/tpc.109.067934 PMC2861458

[21] Yi, H. et al. Constitutive expression exposes functional redundancy between the Arabidopsis histone H2A gene HTA1 and other H2A gene family members. *Plant. Cell.* **18**, 1575–1589 (2006). 16751347 10.1105/tpc.105.039719 PMC1488917

[22] Lacroix, B., Fratta, A., Hak, H., Hu, Y. & Citovsky, V. *Agrobacterium* virulence factors induce the expression of host DNA repair-related genes without promoting major genomic damage. *Sci. Rep.* **14**, 24330 (2024). 39420028 10.1038/s41598-024-75525-8 PMC11487168

[23] Franklin, G. & Dias, A. C. P. Organogenesis and embryogenesis in several *Hypericum perforatum* genotypes. *Vitro Cell. Dev. Biology - Plant.* **42**, 324–330 (2006).

[24] Pradeep, M. & Franklin, G. Understanding the hypericin biosynthesis via reversible Inhibition of dark gland development in *Hypericum perforatum* L. *Ind. Crops Prod.* **182**, 114876 (2022).

[25] Murashige, T. & Skoog, F. A. Revised medium for rapid growth and bio assays with tobacco tissue cultures. *Physiol. Plant.* **15**, 473–497 (1962).

[26] Kuriakose, B., Ganesan, V., Thomas, G., Viswanathan, A. & Anand, N. Random amplification of genomic ends (RAGE) as an efficient method for isolation and cloning of promoters and uncloned genomic regions. *Afr. J. Biotechnol.* **8**, 4765–4773 (2009).

[27] Madeira, F. et al. The EMBL-EBI job dispatcher sequence analysis tools framework in 2024. *Nucleic Acids Res.* **52**, W521–W525 (2024). 38597606 10.1093/nar/gkae241 PMC11223882

[28] Higo, K., Ugawa, Y., Iwamoto, M. & Korenaga, T. Plant *cis*-acting regulatory DNA elements (PLACE) database: 1999. *Nucleic Acids Res.* **27**, 297–300 (1999). 9847208 10.1093/nar/27.1.297 PMC148163

[29] Lescot, M. et al. PlantCARE, a database of plant *cis*-acting regulatory elements and a portal to tools for in Silico analysis of promoter sequences. *Nucleic Acids Res.* **30**, 325–327 (2002). 11752327 10.1093/nar/30.1.325 PMC99092

[30] Chow, C. N. et al. PlantPAN 2.0: an update of plant promoter analysis navigator for reconstructing transcriptional regulatory networks in plants. *Nucleic Acids Res.* **44**, D1154–D1160 (2016). 26476450 10.1093/nar/gkv1035 PMC4702776

[31] Horsch, R. B. et al. A simple and general method for transferring genes into plants. *Sci. (1979)*. **227**, 1229–1231 (1985). 10.1126/science.227.4691.1229 17757866

[32] Ishka, M. R. et al. Natural variation in salt-induced changes in root:shoot ratio reveals SR3G as a negative regulator of root suberization and salt resilience in Arabidopsis. *Elife* **13**, RP98896 (2024). 10.7554/eLife.98896 PMC11952752 40153306

[33] Kumar, G. & Singh, A. K. Reference gene validation for qRT-PCR based gene expression studies in different developmental stages and under biotic stress in Apple. *Sci. Hortic.* **197**, 597–606 (2015).

[34] Livak, K. J. & Schmittgen, T. D. Analysis of relative gene expression data using Real-Time quantitative PCR and the 2^–∆∆CT^ method. *Methods* **25**, 402–408 (2001). 11846609 10.1006/meth.2001.1262

[35] Jin, J. et al. PlantTFDB 4.0: toward a central hub for transcription factors and regulatory interactions in plants. *Nucleic Acids Res.* **45**, D1040–D1045 (2017). 27924042 10.1093/nar/gkw982 PMC5210657

[36] Kong, W., Ding, L., Cheng, J. & Wang, B. Identification and expression analysis of genes with pathogen-inducible *cis*-regulatory elements in the promoter regions in Oryza sativa. *Rice* **11**, 52 (2018). 30209707 10.1186/s12284-018-0243-0 PMC6135729

[37] Javed, T. & Gao, S. J. WRKY transcription factors in plant defense. *Trends Genet.* **39**, 787–801 (2023). 37633768 10.1016/j.tig.2023.07.001

[38] Wyrsch, I., Domínguez-Ferreras, A., Geldner, N. & Boller, T. Tissue-specific FLAGELLIN-SENSING 2 (FLS2) expression in roots restores immune responses in Arabidopsis fls2 mutants. *New Phytol.* **206**, 774–784 (2015). 25627577 10.1111/nph.13280

[39] Li, N. et al. OsASR2 regulates the expression of a defence-related gene, Os2H16, by targeting the GT-1 *cis*-element. *Plant. Biotechnol. J.* **16**, 771–783 (2018). 28869785 10.1111/pbi.12827 PMC5814579

[40] Košuth, J., Katkovčinová, Z., Olexová, P. & Čellárová, E. Expression of the hyp-1 gene in early stages of development of *Hypericum perforatum* L. *Plant. Cell. Rep.* **26**, 211–217 (2007). 16988829 10.1007/s00299-006-0240-4

[41] Z Freitas, F. et al. The SEB-1 transcription factor binds to the STRE motif in neurospora crassa and regulates a variety of cellular processes including the stress response and reserve carbohydrate metabolism. *G3 Genes|Genomes|Genetics*. **6**, 1327–1343 (2016). 26994287 10.1534/g3.116.028506 PMC4856084

[42] Singh, K. B. & Foley, R. C. Oñate-Sánchez, L. Transcription factors in plant defense and stress responses. *Curr. Opin. Plant. Biol.* **5**, 430–436 (2002). 12183182 10.1016/s1369-5266(02)00289-3

[43] Estruch, F. Stress-controlled transcription factors, stress-induced genes and stress tolerance in budding yeast. *FEMS Microbiol. Rev.* **24**, 469–486 (2000). 10978547 10.1111/j.1574-6976.2000.tb00551.x

[44] Vihervaara, A. et al. Transcriptional response to stress is pre-wired by promoter and enhancer architecture. *Nat. Commun.* **8**, 255 (2017). 28811569 10.1038/s41467-017-00151-0 PMC5557961

[45] Rabeh, K., Hnini, M. & Oubohssaine, M. A comprehensive review of transcription factor-mediated regulation of secondary metabolites in plants under environmental stress. *Stress Biology*. **5**, 15 (2025).

[46] Tzfira, T., Vaidya, M. & Citovsky, V. VIP1, an *Arabidopsis* protein that interacts with *Agrobacterium* VirE2, is involved in VirE2 nuclear import and *Agrobacterium* infectivity. *EMBO J.* **20**, 3596–3607 (2001). 11432846 10.1093/emboj/20.13.3596 PMC125502

[47] Lacroix, B. & Citovsky, V. Characterization of VIP1 activity as a transcriptional regulator in vitro and in planta. *Sci. Rep.* **3**, 2440 (2013). 23942522 10.1038/srep02440 PMC3743055

[48] Pitzschke, A., Djamei, A., Teige, M. & Hirt, H. VIP1 response elements mediate mitogen-activated protein kinase 3-induced stress gene expression. *Proc. Natl. Acad. Sci.* **106**, 18414–18419 (2009). 19820165 10.1073/pnas.0905599106 PMC2759709

[49] Gao, Y. et al. Pseudomonas syringae activates ZAT18 to inhibit Salicylic acid accumulation by repressing EDS1 transcription for bacterial infection. *New Phytol.* **233**, 1274–1288 (2022). 34797591 10.1111/nph.17870

[50] Anand, A. et al. Salicylic acid and systemic acquired resistance play a role in attenuating crown gall disease caused by *Agrobacterium tumefaciens*. *Plant. Physiol.* **146**, 703–715 (2008). 18156296 10.1104/pp.107.111302 PMC2245820

[51] Ullah, C., Chen, Y. H., Ortega, M. A. & Tsai, C. J. The diversity of Salicylic acid biosynthesis and defense signaling in plants: knowledge gaps and future opportunities. *Curr. Opin. Plant. Biol.* **72**, 102349 (2023). 36842224 10.1016/j.pbi.2023.102349

[52] Niemi, O. et al. Genome sequence of the model plant pathogen *Pectobacterium carotovorum* SCC1. *Stand. Genomic Sci.* **12**, 87 (2017). 29276572 10.1186/s40793-017-0301-z PMC5738896

[53] Falak, N., Imran, Q. M., Hussain, A. & Yun, B. W. Transcription factors as the blitzkrieg of plant defense: A pragmatic view of nitric oxide’s role in gene regulation. *Int J. Mol. Sci* **22**, (2021). 10.3390/ijms22020522 PMC7825681 33430258

[54] Hong, J. K. & Hwang, B. K. The promoter of the pepper pathogen-induced membrane protein gene CaPIMP1 mediates environmental stress responses in plants. *Planta* **229**, 249–259 (2009). 18936963 10.1007/s00425-008-0824-z

[55] Jia, X. et al. The origin and evolution of Salicylic acid signaling and biosynthesis in plants. *Mol. Plant.* **16**, 245–259 (2023). 36476805 10.1016/j.molp.2022.12.002

[56] Kesarwani, M., Yoo, J. & Dong, X. Genetic interactions of TGA transcription factors in the regulation of Pathogenesis-Related genes and disease resistance in Arabidopsis. *Plant. Physiol.* **144**, 336–346 (2007). 17369431 10.1104/pp.106.095299 PMC1913812

[57] Mishra, S. et al. Salicylic acid (SA)-mediated plant immunity against biotic stresses: an insight on molecular components and signaling mechanism. *Plant. Stress*. **11**, 100427 (2024).

[58] Mou, Z., Fan, W. & Dong, X. Inducers of plant systemic acquired resistance regulate NPR1 function through redox changes. *Cell* **113**, 935–944 (2003). 12837250 10.1016/s0092-8674(03)00429-x

[59] F Klessig, D. et al. Nitric oxide and Salicylic acid signaling in plant defense. *Proc. Natl. Acad. Sci.* **97**, 8849–8855 (2000). 10922045 10.1073/pnas.97.16.8849 PMC34022

[60] Ryu, H. et al. Flagellin Sensing, Signaling, and immune responses in plants. *Plant. Commun.*10.1016/j.xplc.2025.101383 (2025). 40400167 10.1016/j.xplc.2025.101383 PMC12281300

