# Supplementary material for: Transgenic validation of a promoter strongly inducible by Agrobacterium tumefaciens
Source: Sci Rep. 2025 Dec 1;16:485. doi: 10.1038/s41598-025-30002-8 (PMC12775061; doi:10.1038/s41598-025-30002-8)
Supplement: Supplementary file 1 — Supplementary Material 1 [file 41598_2025_30002_MOESM1_ESM.docx]

Supplementary material

**Transgenic validation of a promoter strongly inducible by *Agrobacterium tumefaciens***

Rakesh Sinha, Preeti Shakya, Rajendran K. Selvakesavan, Gregory Franklin^*^

Institute of Plant Genetics of the Polish Academy of Sciences, Strzeszyńska 34, 60-479

Poznań, Poland

Fig. S1. Quantitative real-time PCR analysis of *hyp1* gene expression in *Hypericum perforatum* cell suspension cultures after co-cultivation with *Agrobacterium tumefaciens* at different time points (0.5–24 h)

>Full-length Promoter (HyPRO)

**AAAATCCAGGGGCGTACGTACGATCGGAAGAGTGTTGTTCCAGACTTAATGCGGTTCCTGTGTTTACATATGTGCACATTTAGAATTTCCTCTACGAGATTGCGACCACGCACCTTATTCAAAATCGTGCGACGGTTTTAAGAAAACCGCAAAAAAAAAAAAAATTGTGATAGGATTATTAGATCACAACTCTACACCGCATATCTCCAAAATACATAAACCGTATCAACACCTCTAACACCTATAATTGCAAACGCAGTCAAAGTGCAGCGGTCACCCATCAAGCACATTAAATAGAAGTCCCTTTTACCAACCACCTTGCTGGCTCATTAAACGTGAAGCCCATCTCCTTGGAACAACTCGCACTAATTAGCTCCTTTATAAGAGGTAAATTGGCTACAAATTATAAACTTTAAGCAATTAATTAGCTTTTGTAGAATCGAGACTCGTTAACGTTAAAGCAAATTTCAGAGTTTGACAATTTTTTTCCCTCTATTAAATGGTCATGATGTCCTGTCGGTGTAGATTCATTGTACGTAGTTAATGATTTTTTTATGATACATAGGTTAGTTCTTTAGCCAAATTGCTTATACAGCAACATAGGTCAACAACCGGATAAGATATAAACTTACAAAAAACCGAATAAGATATAAGTAAGTGAAACTATTAGTGTATTAATTTTACACGAAAAAACTTTTCCACTTACATTGTATGATATTTATCCAAACACAATTTTTTATTAGTACACAAATTATATTTTATTTCTCGGGCAAGGATTAATATGACTCGTTTAGTGGGAGGCATTCATAATAGAAGTGATAGGCTGTGACAGCGAATGAGAGACATAGTTTATATTTCAGGGGACGATATTATTTTTTAGACGGTTTCTAGAAGATGAGACTTGGCTCGATTATTCAACAATGGAGTGCTGGTTTCAGATTGAAAATTTAGAGGCCAATAACTTTCTTAGCCCTATAAATAGCGACCTTCTAAGTCTGTTTCGTCACAACACAAAATACAGATACATTAGTTAGTGATCAGTGTTTGTATACTTTCTCATCTTTAGCTATTTTAACATTTCTGAAT**

>HyPRO-TR1

**~~AAAATCCAGGGGCGTACGTACGATCGGAAGAGTGTTGTTCCAGACTTAATGCGGTTCCTGTGTTTACATATGTGCACATTTAGAATTTCCTCTACGAGATTGCGACCACGCACCTTATTCAAAATCGTGCGACGGTTTTAAGAAAACCGCAAAAAAAAAAAAAATTGTGATAGGATTATTAGATCACAACTCTACACCGCATATCTCCAAAATACATAAACCGTATCAACACCTCTAACACCTATAATTGCAAACGCAGTCAAAGTGCAGCGGTCACCCATCAAGCACATTAAATAGAAGTCCCTTTTACCAACCACCTTGCTGGCTCATTAAACGTGAAGCCCATCTCCTTGGAACA~~ACTCGCACTAATTAGCTCCTTTATAAGAGGTAAATTGGCTACAAATTATAAACTTTAAGCAATTAATTAGCTTTTGTAGAATCGAGACTCGTTAACGTTAAAGCAAATTTCAGAGTTTGACAATTTTTTTCCCTCTATTAAATGGTCATGATGTCCTGTCGGTGTAGATTCATTGTACGTAGTTAATGATTTTTTTATGATACATAGGTTAGTTCTTTAGCCAAATTGCTTATACAGCAACATAGGTCAACAACCGGATAAGATATAAACTTACAAAAAACCGAATAAGATATAAGTAAGTGAAACTATTAGTGTATTAATTTTACACGAAAAAACTTTTCCACTTACATTGTATGATATTTATCCAAACACAATTTTTTATTAGTACACAAATTATATTTTATTTCTCGGGCAAGGATTAATATGACTCGTTTAGTGGGAGGCATTCATAATAGAAGTGATAGGCTGTGACAGCGAATGAGAGACATAGTTTATATTTCAGGGGACGATATTATTTTTTAGACGGTTTCTAGAAGATGAGACTTGGCTCGATTATTCAACAATGGAGTGCTGGTTTCAGATTGAAAATTTAGAGGCCAATAACTTTCTTAGCCCTATAAATAGCGACCTTCTAAGTCTGTTTCGTCACAACACAAAATACAGATACATTAGTTAGTGATCAGTGTTTGTATACTTTCTCATCTTTAGCTATTTTAACATTTCTGAAT**

>HyPRO-TR2

**~~AAAATCCAGGGGCGTACGTACGATCGGAAGAGTGTTGTTCCAGACTTAATGCGGTTCCTGTGTTTACATATGTGCACATTTAGAATTTCCTCTACGAGATTGCGACCACGCACCTTATTCAAAATCGTGCGACGGTTTTAAGAAAACCGCAAAAAAAAAAAAAATTGTGATAGGATTATTAGATCACAACTCTACACCGCATATCTCCAAAATACATAAACCGTATCAACACCTCTAACACCTATAATTGCAAACGCAGTCAAAGTGCAGCGGTCACCCATCAAGCACATTAAATAGAAGTCCCTTTTACCAACCACCTTGCTGGCTCATTAAACGTGAAGCCCATCTCCTTGGAACAACTCGCACTAATTAGCTCCTTTATAAGAGGTAAATTGGCTACAAATTATAAACTTTAAGCAATTAATTAGCTTTTGTAGAATCGAGACTCGTTAACGTTAAAGCAAATTTCAGAGTTTGACAATTTTTTTCCCTCTATTAAATGGTCATGATGTCCTGTCGGTGTAGATTCATTGTACGTAGTTAATGATTTTTTTATGATACATAGGTTAGTTCTTTAGCCAAATTGCTTATACAGCAA~~CATAGGTCAACAACCGGATAAGATATAAACTTACAAAAAACCGAATAAGATATAAGTAAGTGAAACTATTAGTGTATTAATTTTACACGAAAAAACTTTTCCACTTACATTGTATGATATTTATCCAAACACAATTTTTTATTAGTACACAAATTATATTTTATTTCTCGGGCAAGGATTAATATGACTCGTTTAGTGGGAGGCATTCATAATAGAAGTGATAGGCTGTGACAGCGAATGAGAGACATAGTTTATATTTCAGGGGACGATATTATTTTTTAGACGGTTTCTAGAAGATGAGACTTGGCTCGATTATTCAACAATGGAGTGCTGGTTTCAGATTGAAAATTTAGAGGCCAATAACTTTCTTAGCCCTATAAATAGCGACCTTCTAAGTCTGTTTCGTCACAACACAAAATACAGATACATTAGTTAGTGATCAGTGTTTGTATACTTTCTCATCTTTAGCTATTTTAACATTTCTGAAT**

Fig. S2. Complete sequence of the HyPRO promoter


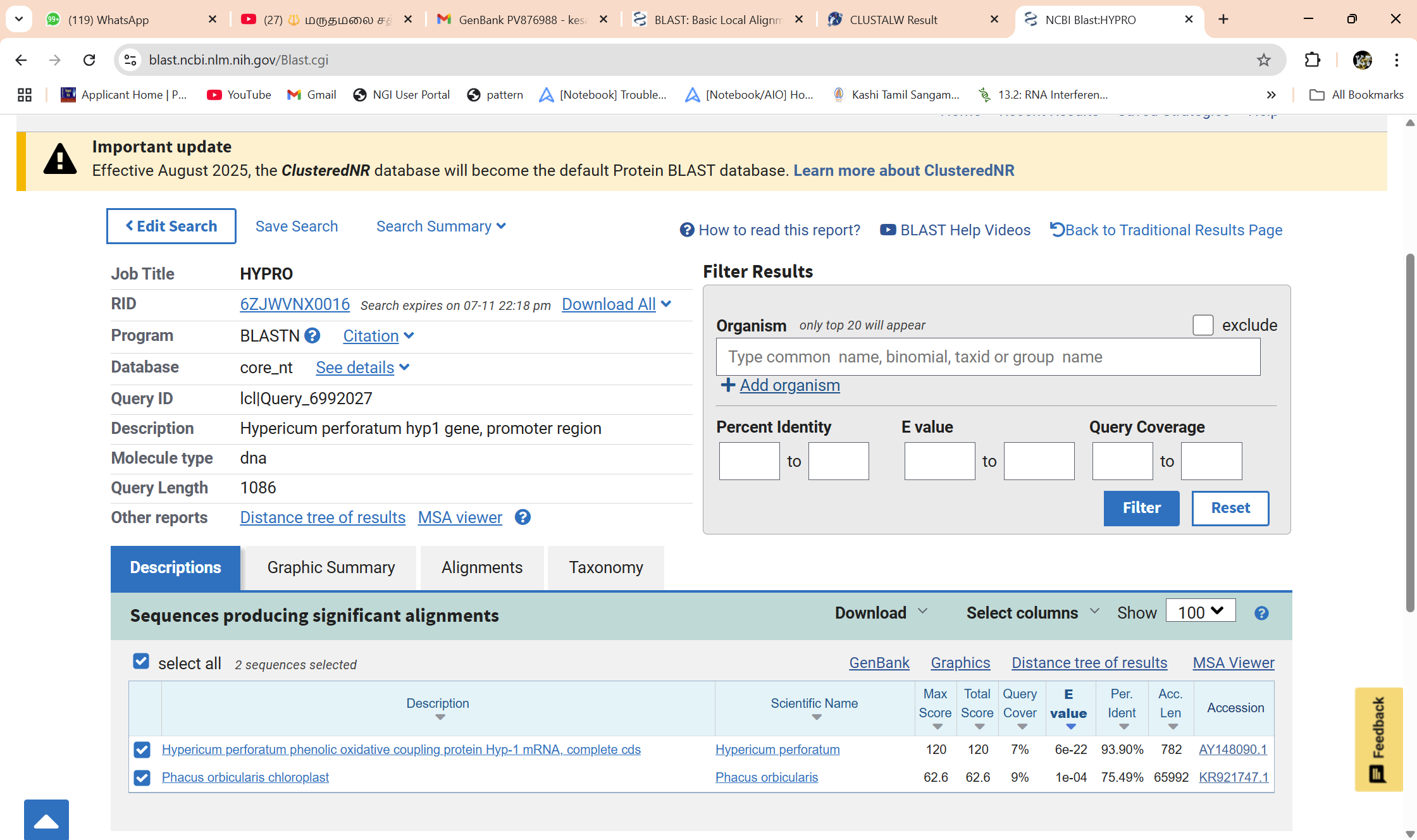


Fig S3. BLAST analysis of full-length promoter

Table S1. List of primers used for amplification of truncated promoter fragments and for qRT-PCR analysis

| Primer Name | Sequence (5-3) |
| --- | --- |
| HYP1TR1F | CACCACTCGCACTAATTAGCTCCT |
| HYP1TR2F | CACCCATAGGTCAACAACCGGATA |
| HYP1R | ATTCAGAAATGTTAAAATAGCTAAAGA |
| Hyp-F | GTGCACACTAGTATGGCGGCGTACACTATT |
| Hyp-R | gtgcacgagctcttaagcgaaaacttcagg |
| GUS F | GATCGCGAAAACTGTGGAAT |
| GUS R | TGAGCGTCGCAGAACATTAC |
| GAPDH F | CAAGCCAGACATCCACATTCTTT |
| GAPDH R | ACCCTCAACAATTCCGAACCT |

Table S2. Putative *cis-*regulatory elements of full-length promoter predicted by online tools (PlantCARE, PLACE and PlantPAN)

| *cis*-element name | Position | Function | HyPRO (1116) | HyPRO-TR1 (728 bp) | HyPRO-TR2 (488 bp) |
| --- | --- | --- | --- | --- | --- |
| **Category: Abiotic stress Responsive elements** | | |  |  |  |
| Box 4 | -667, -311, -413 | Part of a conserved DNA module involved in light responsiveness | 3 | 3 | 2 |
| CCAATBOX1 | -694, -131 | Heat/stress responsive elements | 2 | 2 | 2 |
| MYBCORE | -477 | Drought stress-responsive element | 1 | 1 | 1 |
| ABRE | -753 | ABA responsive element | 2 | 1 | 0 |
| ARE | -157 | Anaerobic induction responsive element | 1 | 1 | 1 |
| LTREATLTI78, LTRECOREATCOR15 | -571, -572 | Cold responsive element | 2 | 2 | A |
| DRE2COREZMRAB17, | -570 | Dehydration-responsive element | 1 | 1 | A |
| STRE | -1079, -228 | stress responsive element, core promoter element around -30 of transcription start | 2 | 1 | 1 |
| MYB | -776 | involves in hormone signal transduction, and abiotic stress tolerance | 1 | A | A |
| TC-rich repeats | -949 | Plant defense responsive elements | 1 | A | A |
| ABRELATERD1 | -752 | ABRE-related sequence | 1 | A | A |
| MYCCONSENSUSAT | -1019 | MYC elements related to dehydration responses. | 1 | A | A |
| CBFHV | -570 | Cold responsive elements | 1 | 1 | A |
| GT1GMSCAM4 | -399, -602 | Salt stress and Light-responsive element | 2 | 2 | 1 |
| CURECORECR | -343, +1072, -1068 | Oxygen and Cu responsive element | 3 | 1 | 1 |
| CuRE | -340, -550, -1073,  -548, -630, -749, -1067 | Copper signalling responsive element | 7 | 4 | 1 |
| MYBCORE | +477 | Water stress responsive element | 1 | 1 | 1 |
| MYBST1 | -366, -472 | Transcription activator | 2 | 2 | 2 |
| IBOX | -471, -367 | Light-responsive element | 2 | 2 | 2 |
| MYB1AT | -156 | Draught and ABA-induced responsive element | 1 | 1 | 1 |
| WBOXHVISO1 | -828, -303 | Sugar-responsive element | 2 | 1 | 1 |
| PYRIMIDINEBOXOSRAMY1A | -783 | Sugar-responsive element | 2 | A | A |
| G-Box | -754, -756 | light responsive element | 2 | A | A |
| GATA-motif, GATABOX | -918, -466, -439, -372, -366, -268, -217, -62, | Light responsive element | 8 | 7 | 7 |
| WBOXATNPR1 | -611, -482, -827 | Light responsive elements | 3 | 2 | 1 |
| PRECONSCRHSP70A | -1082 | Plastid, light and HSP gene responsive element | 1 | A | A |
| HSP26 | -288 | HSP gene induction responsive element | 1 | 1 | 1 |
| ACGTATERD1 | -1070, -752, -633, -551 | Dehydration responsive element | 4 | 2 | A |
| TATABOXOSPAL | -796 | Plant defense responsive elements | 1 | A | A |
| MYB1LEPR | -520 | Plant defense responsive elements | 1 | 1 | A |
| AT1-motif | -147 | Light responsive elements | 1 | 1 | 1 |
| SURECOREATSULTR11 | -644, -246, -189 | Sulfur-responsive element | 3 | 3 | 2 |
| BOXIINTPATPB | -792, -276 | Abiotic stress responsive elements | 2 | 1 | 1 |
| ACGTABOX | -1071, -552 | A-Box, sugar metabolism responsive elements | 2 | 1 | A |
| MYCCONSENSUSAT | -1019 | Cold and dehydration responsive element | 1 | A | A |
| ACGTTBOX | -634 | Dehydration-responsive (DRE) element | 1 | 1 | A |
| TATABOX2 | -112 | Primary metabolism: light responsive elements | 1 | 1 | 1 |
| **Category: Phytohormones** | | |  |  |  |
| CGTCA-motif | -85 | MeJA-responsive elements | 1 | 1 | 1 |
| TGACG-motif | -85 | MeJA-responsive elements | 1 | 1 | 1 |
| CAREOSREP1 | -729 | IAA and GA-responsive elements. | 1 | A | A |
| GE | -825, -895 | GA responsive elements | 2 | A | A |
| SEBFCONSSTPR10A | -856, -851 | Auxin responsive element | 2 | A | A |
| ARFAT | -260 | Phytohormone responsive element | 1 | 1 | 1 |
| T/GBOXATPIN2 | -246 | JA-responsive element | 1 | 1 | 1 |
| GT1CONSENSUS | -753 | SA-inducible PR gene expression, SAR | 1 | A | A |
| ARR1AT | -1001, -780, -699, -399, --602, -391, -368, -144 | Cytokinin response elements | 10 | 6 | 4 |
| DPBFCOREDCDC3 | -1083 | ABA response elements | 1 | 1 | 1 |
| CATATGGMSAUR | -1029 | Auxin responsive elements | 1 | 1 | 1 |
| CPBCSPOR | -1019, -909, -471, -348 | Ethylene, Cytokinin-enhanced Protein Binding site | 4 | 2 | 2 |
| ELRECOREPCRP1 | -483 | Salicylic acid/ elicitor responsive element. | 1 | 1 | 1 |
| WBBOXPCWRKY1 | -828 | Pathogen/elicitor responsive element. | 1 | A | A |
| AP2; ERF | -544, -544, -386, -275, -600 | TFs binding site and elicitor-responsive element | 5 | 5 | 2 |
| **Category: Tissue/cell specific and Development-Related** | | |  |  |  |
| RHERPATEXPA7 | -961 | Root hair-specific elements | 1 | A | A |
| MARTBOX | -935, -932, -933, -934 | Mesophyll-specific elements | 4 | A | A |
| CACTF TPPCA1 | -1055, -344, -293, -161, -53, -46, -36 | Mesophyll-specific elements | 7 | 6 | 6 |
| RAV1, RAVAAT | -855, -485, -476, -166, -79, -1049 | Rosette leaves and roots specific elements | 6 | 4 | 4 |
| GTGANTG10  QELEMENTZMZM13 | -919, -750, -428, -846, -856, -270, -260, -842, -484, -812, -82, -52 | Pollen-specific elements | 12 | 6 | 6 |
| DOFCOREZM | -824, -628, -513, -392, -124, -34, -25 | Mesophyll-specific elements | 6 | 6 | 3 |
| SITEIIATCYTC | -370 | Meristem-specific element | 1 | 1 | 1 |
| TAAAGSTKST1 | -709 | Guard cell specific element | 1 | A | A |
| 300ELEMENT | -408 | Pollen and embryos specific element | 1 | 1 | 1 |
| POLLEN1LELAT52 | -33, -8 | Pollen specific element | 2 | 2 | 2 |
| MYBPZM | -776 | Secondary metabolic pathways responsive elements | 1 | A | A |
| Root motif TA Pox1 | -307, -234, -239 | Root-specific elements | 3 | 3 | 3 |
| **Category: Transcription Factors and other motifs** | | |  |  |  |
| NAC; NAM | -562 | NAC-domain TFs binding site | 1 | 1 | A |
| Myb/SANT | -250, -742, -228, -648 | TFs binding site | 4 | 3 | 2 |
| HSF | -201 | TFs binding site | 1 | 1 | 1 |
| bZIP | -558 | TFs binding site | 1 | 1 | A |
| Dof | -18, -633 | TFs binding site | 2 | 2 | 1 |
| bHLH | -1072 | TF binding site | 1 | A | A |
| C2H2 | -47, -695, | TF binding site | 2 | 2 | 1 |
| EIN3; EIL | -67, -531 | TF binding site | 1 | 2 | 1 |
| GATA; tify | -906, -53, -752 | TF binding site | 3 | 1 | 1 |
| TALE | -261 | TF binding site | 1 | 1 | 1 |
| MADF | -638, | TF binding site | 1 | 1 | A |
| Myb/SANT | -117, -563 | TF binding site | 2 | 2 | 1 |
| WRKY | -829, -484 | TF binding site | 2 | 1 | 1 |
| CAAT-box | -988, -504, -625, -168, -687, -357, -507, -132, -840, -380, -608, -148, -669, -339, -557, -131, -923, -695 | common CREs and TFs binding site | 18 | 15 | 7 |
| CAATBOX1 | -839, -694, -607, -556, -499, -377, -356, -147, -130 | TF s binding site | 9 | 8 | 4 |
| TATABOX3 | -413, -310 | TATA box | 2 | 2 | 2 |
| TATCCAOSAMY | -366 | Alpha-amylase, MYB proteins, GA, responsive elements | 1 | 1 | 1 |
| LECPLEACS2 | -332 | Cys-protease binding element | 1 | 1 | 1 |
